# Supplementary material for: Individual and Simultaneous Photodegradation of Trimethoprim and Sulfamethoxazole Assessed with the Microbial Assay for Risk Assessment
Source: Molecules. 2025 Apr 25;30(9):1907. doi: 10.3390/molecules30091907 (PMC12073508; doi:10.3390/molecules30091907)
Supplement: Supplementary file 1 [file molecules-30-01907-s001.zip › molecules-3571635-supplementary.pdf]

Operating Manual SUNTEST® CPS+

ATLAS MATERIAL TESTING SOLUTIONS

3 Description of the instrument

3.3 Test chamber

- Door lock switch ① for opening / closing the test chamber door ②
- The reflector ③ is located in the test chamber and in the test chamber door. The xenon lamp is located in the roof of the test chamber
- The sensor ④ to measure the irradiance (E)
- Black standard sensor (BST) ⑥ to measure the black standard temperature at sample level
- Air inlet ⑤ for temperature control in the test chamber
- Sample table ⑦ used for positioning the samples

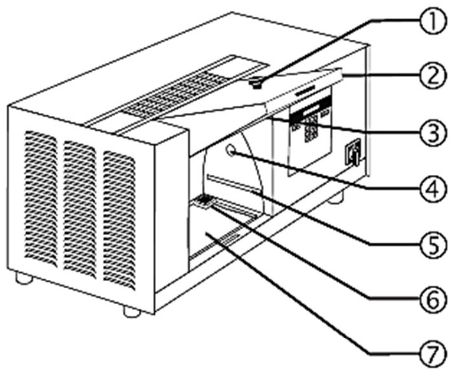

① Door lock switch
② Test chamber door
③ Reflector
④ Sensor for measuring irradiance
⑤ Air inlet for temperature regulation in the test chamber
⑥ Black standard sensor (BST)
⑦ Sample table

Fig. 3

Figure S1. The diagram of the SunTest CPS+ apparatus.
(www. .manualslib.com/manual/1516376/Atlas-Suntest-CpsPlus.html)
